# Augmented Reality in Spinal Surgery: Highlights From Augmented Reality Lectures at the Emerging Technologies Annual Meetings

**DOI:** 10.7759/cureus.19165

**Published:** 2021-10-31

**Authors:** Syed-Abdullah Uddin, George Hanna, Lindsey Ross, Camilo Molina, Timur Urakov, Patrick Johnson, Terrence Kim, Doniel Drazin

**Affiliations:** 1 School of Medicine, University of California Riverside, Riverside, USA; 2 Neurosurgery, Cedars-Sinai Spine Center, Los Angeles, USA; 3 Neurology and Neurosurgery, Cedars-Sinai Medical Center, Los Angeles, USA; 4 Neurological Surgery, Washington University School of Medicine, St. Louis, USA; 5 Neurological Surgery, University of Miami, Miami, USA; 6 Neurological Surgery, Cedars-Sinai Medical Center, Los Angeles, USA; 7 Orthopedic Surgery, Cedars-Sinai Medical Center, Los Angeles, USA; 8 Medicine, Pacific Northwest University of Health Sciences, Yakima, USA

**Keywords:** augmented reality, mixed reality, spine surgery, orthopedic surgery, neurological surgery, virtual reality

## Abstract

Introduction

Augmented reality (AR) is an advanced technology and emerging field that has been adopted into spine surgery to enhance care and outcomes. AR superimposes a three-dimensional computer-generated image over the normal anatomy of interest in order to facilitate visualization of deep structures without the ability to directly see them.

Objective

To summarize the latest literature and highlight AR from the annual “Spinal Navigation, Emerging Technologies and Systems Integration” meeting lectures presented by the Seattle Science Foundation (SSF) on the development and use of augmented reality in spinal surgery.

Methods

We performed a comprehensive literature review from 2016 to 2020 on PubMed to correlate with lectures given at the annual “Emerging Technologies” conferences. After the exclusion of papers that concerned non-spine surgery specialties, a total of 54 papers concerning AR in spinal applications were found. The articles were then categorized by content and focus.

Results

The 54 papers were divided into six major focused topics: training, proof of concept, feasibility and usability, clinical evaluation, state of technology, and nonsurgical applications. The greatest number of papers were published during 2020. Each paper discussed varied topics such as patient rehabilitation, proof of concept, workflow, applications in neurological and orthopedic spine surgery, and outcomes data.

Conclusions

The recent literature and SSF lectures on AR provide a solid base and demonstrate the emergence of an advanced technology that offers a platform for an advantageous technique that is superior, in that it allows the operating surgeon to focus directly on the patient rather than a guidance screen.

## Introduction

The concept of using the navigation in performing more accurate cranial and spinal procedures extends back to the novel concept of using fluoroscopy and television technology to perform a stereotactic cordotomy in the 1960s [1]. Cranial neuronavigation has become the standard of care and has gained widespread usage in spine surgery. The incorporation of image guidance has been transformative in spine surgery and has increased the accuracy and safety of complex spinal procedures [2,3]. One of the earliest feasibility studies evaluating the use of augmented reality (AR) in a spine phantom demonstrated reproducibility and the usefulness of displaying projections to assist in navigating complex areas of anatomy [4]. The concept of AR stems from the desire to enhance surgical efficacy, safety, and improve outcomes. Some early clinical studies demonstrated efficacy and safety in areas of spine surgery such as intradural tumor surgery [5] and degenerative spine pathology [6]. AR uses integrated computer and camera technology to superimpose a three-dimensional computer-generated image over the normal anatomy of interest in order to facilitate procedures and provide highlighting of structures of interest without direct visualization, where one is working. This has the advantage of allowing the surgeon to focus their vision on the area of surgery rather than looking at another screen or projection. This has gained further interest with the recent FDA approval of the first AR system in spinal surgery, xVision (Augmedics, Chicago, IL).

The Seattle Science Foundation’s (SSF) annual “Spinal Navigation, Emerging Technologies and Systems Integration” meeting highlights emerging and advancing technologies in multiple disciplines of spinal surgery including intraoperative navigation and robotic surgery. The conference, now in its fifth year, has historically brought together surgeons inspired to incorporate novel technologies in the operating room and surgeons reporting the progress of the technology in their own clinical practices. The goal of the meeting is to talk about the benefits of new technologies for surgeons and patients, encourage further development of these technologies in the field, and discuss adoption across multiple institutions. This series of highlights will examine the use of augmented reality throughout the last five years of the conferences and offer insights into the future direction of the use of these technologies.

## Materials and methods

A PubMed search was carried out with these search terms: augmented reality spine surgery, augmented reality neurosurgery, augmented reality orthopedic surgery, augmented reality surgery, mixed reality spine surgery, OR augmented reality spine surgery. Results were limited to 2016 through 2020, so as to correlate with lectures given at the SSF “Annual Emerging Technologies” conferences. The literature review resulted in 132 papers. Inclusion criteria were defined as papers pertaining directly to neurosurgical or orthopedic spine surgery and published within the 2016-2020 time frame. After the exclusion of papers related to non-spine surgery specialties, there were a total of 54 papers concerning AR in the field of spinal surgery. We then categorized by content and focus of each article.

Following the review of the literature, the agendas of the annual “Spinal Navigation, Emerging Technologies, and Systems Integration” meeting were reviewed for lectures focused on the topic of AR. Six lectures were given across the 2016-2020 time period ranging from introductions to the technology to direct clinical applications and outcomes using FDA-approved AR systems. Lectures were reviewed and annotated into a series of highlights documenting the growth and evolution of AR over the past four years.

## Results

**Figure 1:**
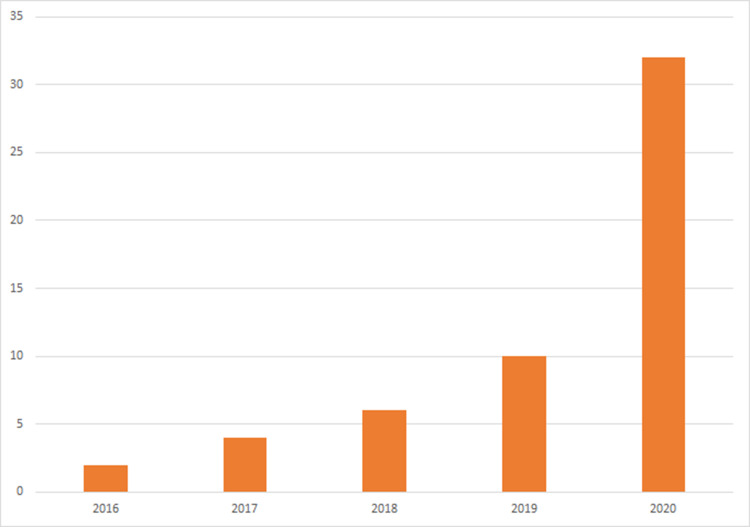
AR spine literature assorted by the year 2016-2020. AR: augmented reality.

Of the 54 papers identified in the literature review with queried terms that pertained to spine surgery, there was a steady increase of AR papers published per queried year, with 2 published in 2016, 4 published in 2017, 6 published in 2018, 10 published in 2019 and 31 published in 2020 (Figure 1).

**Figure 2:**
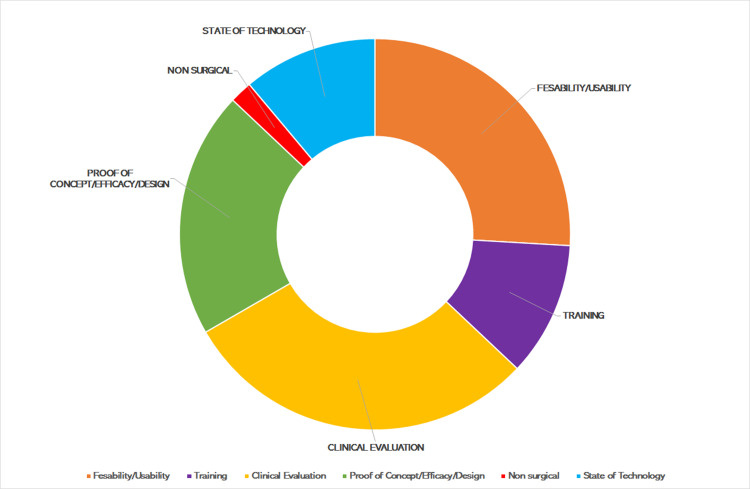
AR spine literature organized by thematic content. AR: augmented reality.

The published AR papers were assessed and categorized by primary focus to include the themes of (1) Training, (2) Proof of Concept, (3) Feasibility & Usability, (4) Clinical Evaluation, (5) State of Technology, and (6) Nonsurgical Applications (Figure 2). Non-surgical papers made up 2% of the literature review and focused on the topic of using AR to aid in the rehabilitation of orthopedic patients [7]. The literature detailed the use of AR to enhance surgeon training [8] and aid in simulation of spinal instrumentation in 9% of the review [9]. More recent papers focused on the use of AR in training were enhanced by the FDA clearance of AR platforms. These papers established a workflow for the use of AR in the operating room and compared the benefits and pitfalls of AR with traditional techniques [10]. Eleven percent of the reviewed papers were categorized as State of the Technology. These papers provided an assessment of the current technological environment of AR and offered future directions for creating a compact, highly versatile, portable AR system that can be applied broadly in the fields of Neurosurgery and Orthopedic surgery [11]. Twenty-one percent of the results were studies focusing on the proof of concept of AR. This included testing efficacy in cadaveric models [12-14] as well as comparing the efficacy of pedicle screw instrumentation in AR with the use of navigation assistance or freehand techniques [15,16]. Feasibility/usability studies were defined as investigations into the practicality, strengths and weaknesses of AR in the operating room (OR), this category accounting for 26% of the query. These studies centered around ergonomics, OR footprint, and accuracy of instrumentation while using AR platforms [12,17-19]. The remaining 30%, with a majority published in 2020 (after FDA clearance to use the xVision AR system) emphasized surgeon’s direct clinical application with AR and the surgical experience and outcomes [5,6,18-20].

## Discussion

AR development

With the advent of intra-operative navigational technology, there has been an increase in the accuracy of pedicle screw placement compared to traditional free-hand techniques [21]. Augmented reality, defined as a computer-generated image superimposed onto a real-world field of view (FOV), looks to improve on existing surgical technologies. As AR continues to gain popularity in the field of surgery, preliminary data has begun to show that it is equivalent to traditional non-AR operative methods at a lower cost [22].

The early years of AR

AR, originally adapted from military application in fighter pilot displays, allowed computer-generated images to be superimposed onto the field of view of the operator [4]. In 2016, Dr. Kris Siemionow reviewed this emerging technology of AR and the foothold that AR was finding in spinal surgery. In his review, the limitations of AR intraoperative navigation were discussed, which included: line-of-sight disruptions between the navigation screen and the operative field, unnatural eye/hand coordination that needed to be developed to successfully utilize navigation, disruptive workflows of different tools and overall limitations of using 2-D images on 3-D anatomy.

Within spinal surgery, AR has been shown to improve surgeon’s accuracy, precision and confidence, while also decreasing implant time and tissue dissection [17,23]. In 2016, there were several AR systems that were being prototyped as ‘proof of concept’ to be adapted into spine surgery. The Google Glass (Google, Mountain View, CA) was one of the 1st generation AR lenses and in their study, Yoon et. al. showed that although this system was reviewed favorably by surgeons, operative time did not change and the limited FOV, lack of image overlay and head tracking had a nauseating effect on surgeons [23]. The “projector approach” to AR, in which an image was projected onto a patient’s body to visualize underlying anatomy, had its limitations as well. The limitation of 2-D images projected on a 3-D surface has line of sight (LOS) issues obstructing the image, and the positioning of the surgeon affecting the view has been well reported [23]. The “reflective mirror” technique, in which a computer-generated overlay was displayed onto a reflective glass above the operative field was also in development. This technology was promising due to accurate anatomical localization in three dimensions as well as tracking of the surgeon’s movements, but the system was deemed overly burdensome to be introduced into an OR. Chen et. al. took the reflective mirror technique and adapted it into a head-mounted system which unsuccessfully resulted in a multi-unit system with multiple sensors, calibration steps, cameras and tools that summated to a large navigational error, a lag effect nauseating the surgeon, as well as difficult calibration and limited focal length while operating [24]. The “tablet-based” approach to AR, in which a tablet camera could display AR-pertinent information, was flawed by the lack of 3D imaging, tablet display detracting from operative field, and difficulty of manipulating instruments while viewing through the tablet. Finally, the HoloLens (Microsoft Corp., Redmond, WA), although capable of 3D-imaging and head tracking, was limited in navigational accuracy, processing power, FOV and comfort [25]. Dr. Siemionow discussed that during these early developmental years, the technology was promising, but AR would not be a viable tool that surgeons added to their regular repertoires.

Recent developments

**Figure 3:**
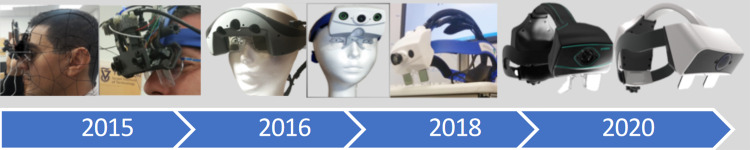
Present timeline demonstrating iterative R&D process of AR, from non-working clinical prototype to live clinical use xVision device. AR: augmented reality.

In 2018, Dr. Camilo Molina discussed a comprehensive AR system that had shown promising results. Highlighting the xVision AR headset, it offered multiple features including a tracking camera mounted directly on the headset (to minimize LOS interruptions), a wireless battery-operated system, an independent navigational system and a series of tool mounts that allowed the xVision system to be agnostic with other manufactured spinal instrumentation systems (Figure 3). Preliminary cadaveric data using xVision in five cadaver torsos was studied with instrumentation from T6-L5 and breach rates graded on the extent and direction of the pedicle screw. The study showed a thoracic screw accuracy rate of 97.1% and a lumbar screw accuracy rate of 96.6% [12]. Compared to traditional freehand screws, reported accuracy of 89%, and manually navigated screws reported accuracy of 96.6%, the xVision system was found to demonstrate equivalent or superior performance [19,20]. xVision AR system began initial steps for clinical use in Israel and was under review by the FDA in the United States.

At the 2019 conference, Dr. Timothy Witham updated the spine community on the developments within the field of AR. At the time of this 2019 talk, the FDA still had not approved the xVision system for commercial use. Dr. Witham began with the Carl et al. study, that had shown the use of AR to superimpose bony landmarks or the outline of tumors on microscopes while operating [26]. It was concluded that AR-assisted microscopic surgery was successful in augmenting a surgeon’s ability to localize anatomy as well as display multiple modes of information to the surgeon [26]. He also referenced Elmi-Terander et al. in their study of a proprietary AR headset that had been used to place pedicle screws in comparison to freehand technique, with regards to accuracy [27]. It was concluded that the use of AR significantly increased precision in placement of screws, while decreasing the incidence of breach rates [27]. At this time, a discussion took place about how surgeons had all reviewed the ease of use and intuitiveness of the AR hardware favorably, stating that the learning curve for all AR systems would be faster than traditional navigation [12]. Additional studies with the xVision system included nine clinical cases in Israel and a cost analysis that suggested AR as a more affordable alternative to traditional navigation.

xVision received FDA clearance in 2020 for clinical use, and Dr. James Lynch spoke of his experience with AR fitting into his practice. Dr. Lynch was the first private practice surgeon to use AR in a community hospital setting within the U.S., with the first documented case being on June 25th, 2020. The workflow was briefly discussed and was found to be similar in setup to spinal navigation with a small ergonomic footprint. At the time of his lecture at “Emerging Technologies,” Dr. Lynch presented his experience with over 50 AR-assisted cases. He demonstrated that AR had led to accurate pedicle screw placements across a variety of instrumentation systems. According to his experience, the learning curve was extremely straightforward with reported proficiency attained at approximately 5 cases. Due to the small ergonomic footprint and a relatively affordable cost of the system, he concluded that the xVision AR system would best find a place in a surgery center where traditional navigation may be too bulky or cost-prohibitive.

The learning curve

**Figure 4:**
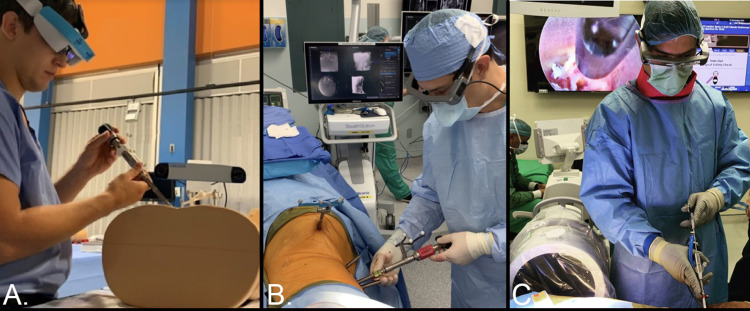
(A) Exploring augmented reality with instrument tracking in the lab (B) utilizing heads-up display as an adjunct to navigated lateral pedicle screw placement and endoscopic diskectomy (C).

With any technology, there is a limitation in the speed of adoption due to novelty and training required to become competent and integrate the system into everyday practice. Dr. Timur Urakov spoke about this process in relation to AR at the 2020 “Emerging Technologies” conference, in which he set out to discuss how AR could be used as an adjunct to already existing technologies instead of being considered an entirely new system workflow (Figure 4). At the University of Miami, Dr. Urakov compared the accuracy of placement of pedicle screws in a cadaveric model. Instrumenting from T1 to pelvis with one side of the cadaver instrumented with fluoroscopy and the contralateral side instrumented with AR assistance, it was found that of the 38 screws placed, fluoroscopy had no breaches while AR-placed screws had 3 major medial breaches and 4 major inferior breaches [14]. The study showed the limitations of current AR systems and suggested that current AR technology should not be used independently, but as an adjunct to already existing technology. His practice then set out to show this concept by 3D-printing adaptors for multiple instrumentation systems and then using them to place screws on sawbones models. Their preliminary data showed that with placement of 60 screws, there were only two low-grade pedicle breaches. Improved accuracy was due to the addition of instrument tracking to the AR system.

The adoption of AR is also more intuitive to those surgeons that use spinal navigation. The visual experience similarities between AR systems and spinal navigation systems are several. With spinal navigation the surgeon is directly viewing the patient’s bony anatomy and instrumentation through a direct-visualization computer screen workstation. The computer screen is typically placed at the head or foot of the bed, away from the patient’s body. AR displays are heads-up displays (HUDs) that show the same information as spinal navigation computer screens, directly onto the surgeon’s AR headset. Dr. Urakov has been able to demonstrate this when he successfully reported on an endoscopic lumbar discectomy with the direct endoscopic images being displayed to the surgeon’s lenses [28]. With direct line of sight of navigation/imaging, surgeons can also apply this technology to more complex cases. Dr. Urakov demonstrated a single-stage lateral/posterior surgery using the Medtronic Stealth Navigation system projecting those images to an AR headset (Figure 4). This allowed the surgeon to fully focus on the operating field instead of shifting focus to a screen away from the operative field multiple times during the case. He concluded that surgeons could maximize their workflow and efficiency by using AR-assisted HUDS to supervise the navigation trajectories of an assistant while working on exposure for a second stage or instrumentation.

Application outside of the OR

Both Drs. Lynch and Urakov emphasized that although AR is promising in the OR, it need not only apply to the spine surgeon with instrumentation. There has been a growing push in health literacy to use tools such as AR to help a patient fully understand the details of their care [29]. Already in the fields of Nephrology and Urology, 3D visualization using AR has shown a higher level of understanding of location, size and surgical options for tumor management [30]. Dr. Urakov has, in his practice, been using AR to show patients their anatomy and explain the details of their case with them. Due to the intuitiveness and ease-of-use with most AR systems, the results have been widely popular among patients, as they get a better understanding (in 3D space) of their pathology and indicated surgical procedures. Dr. Urakov, at the time of his 2020 presentation, with the help of the University of Miami and Magic LeapTM, were working on a patient consultation platform that would convey information primarily through AR. Simulation and education are also possible methods in which AR could be applied to help train the next generation of surgeons. Stefan et al. discuss the use of surgical staff training with traditional and AR-based simulations and the next step forward in medical education [9]. At several residency training centers around the country, surgical residents have been utilizing AR projected HUDS to aid in developing and mastering their surgical coordination and technique. 

Due to the reduced cost compared to navigation, AR may also play a role for surgeons looking to operate in rural parts of the U.S. or even be used on mission trips in resource-limited countries. With traditional navigation computers, cameras and sensors, traditional spinal navigation must be calibrated and present in the operating room for the systems to function properly. This becomes a feasibility issue when sending these systems to areas with limited infrastructure as they may not be able to support these advanced technologies. With further development of AR, a surgeon would simply need to take the AR headset, integrate any imaging modality and use the cross-compatibility seen in multiple AR systems to deploy precise spinal surgery anywhere in the world. 

Future directions 

In 2018, Dr. Molina suggested that AR would be used for minimally invasive applications, and by 2020, there was direct clinical application in Dr. Lynch’s practice in demonstrating that the system lent itself extremely well to accurate placement of pedicle screws in MIS cases.

**Figure 5:**
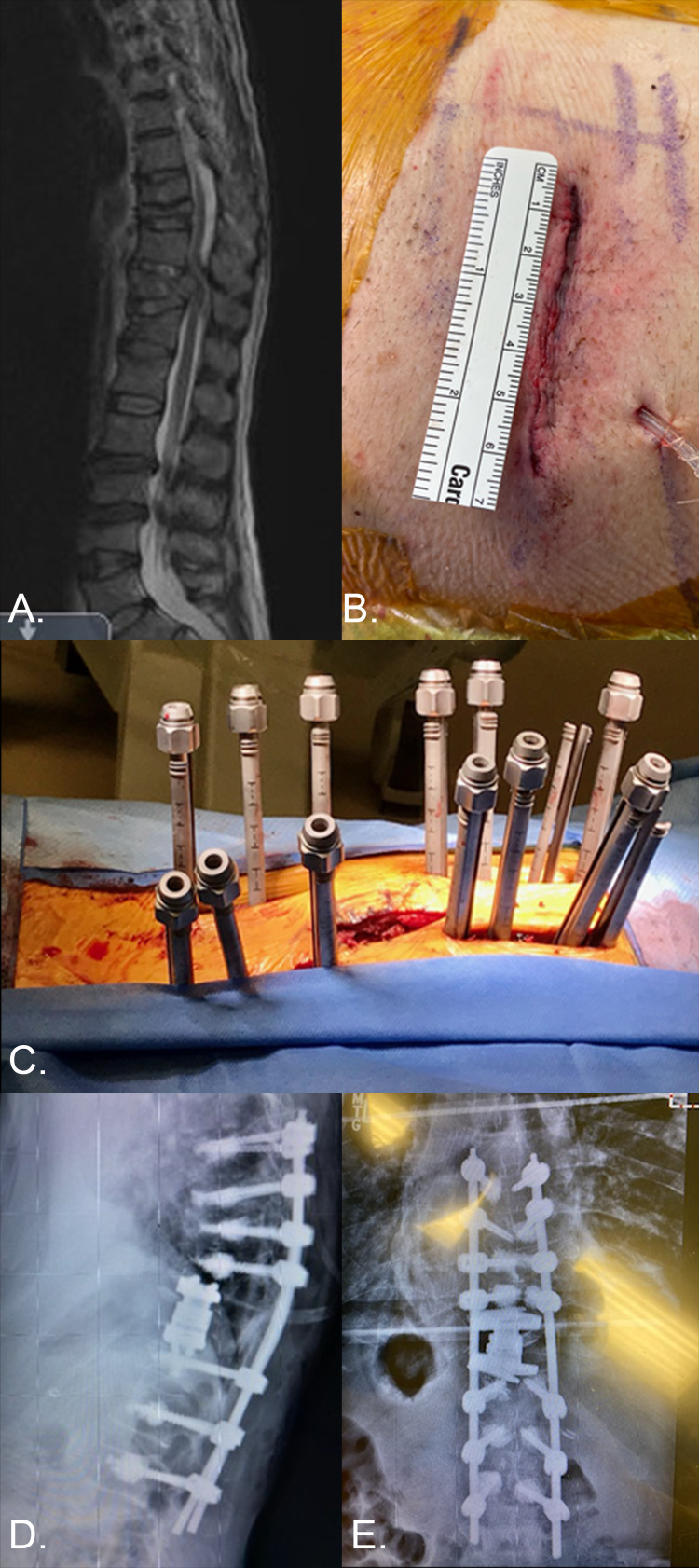
AR case by CAM employing the xvision platform. A. T2W MRI demonstrating T11 pathologic compression fracture resulting in severe spinal canal compression, as well as multiple chronic compression fractures in a 58-year-old myelopathic male with history of lung cancer and severe osteoporosis (T-score:-2.8). B. Stage 1 mini-open retropleural thoracotomy for T11 corpectomy. C,D,E: Stage 2 percutaneous cement augmented T7 - L2 pedicle screw and rod fixation. Case highlights the utility of AR applications in executing technically demanding MIS applications. In this case, the precise insertion of thoracolumbar pedicle screws via AR permitted safe cement augmentation without visceral, vascular, canal, or foraminal cement extravasation. CAM: Camilo A. Molina.

Dr. Molina also expected AR technology to continue to advance to a point where it would be utilized in complex cases such as atrophic/dysplastic bony anatomy, scoliosis with significant coronal and rotational deformities, tumor resection, transarticular screws and even cranial neurosurgery (Figure 5). Dr. Juan Uribe went one step further in his 2020 lecture in which he talked about using AR as a customizable screen to whatever data the surgeon would require. Moving forward from AR, Dr. Uribe stated that “Enhanced Reality” would soon be on the horizon with real-time improvement/modification of surgical images while operating. Dr. Uribe also estimated that the AR market would triple by 2022 with the pioneering AR system, xvision, competing against multiple other AR platforms for a spot in future ORs.

## Conclusions

AR is an emerging technology that has exponentially developed and is continuing to evolve into a superior technology. Its key advantage over robotics and navigated spine surgery is that the surgeon never has to take the focus from the patient. The superimposition of images directly onto the surgical field gives obvious and immediate safety and procedural advantages. As the technology continues to develop, integration of AR should be a tool considered by surgeons parallel to spinal navigation.
